# Efficacy of trabectedin in malignant solitary fibrous tumors: a retrospective analysis from the French Sarcoma Group

**DOI:** 10.1186/s12885-015-1697-8

**Published:** 2015-10-15

**Authors:** J. Khalifa, M. Ouali, L. Chaltiel, S. Le Guellec, A. Le Cesne, J-Y Blay, P. Cousin, L. Chaigneau, E. Bompas, S. Piperno-Neumann, B. Bui-Nguyen, M. Rios, J-P Delord, N. Penel, C. Chevreau

**Affiliations:** 1 Department of Medical Oncology, Institut Claudius Regaud/Institut Universitaire du Cancer de Toulouse – Oncopôle, 1, avenue Irène Joliot-Curie, 31059 Toulouse Cedex 9, France; 2 Department of Statistics, Institut Claudius Regaud / Institut Universitaire du Cancer de Toulouse – Oncopôle, 1, avenue Irène Joliot-Curie, 31059 Toulouse, France; 3 Department of Pathology, Institut Claudius Regaud / Institut Universitaire du Cancer de Toulouse – Oncopôle, 1, avenue Irène Joliot-Curie, 31059 Toulouse, France; 4 Department of Medical Oncology, Institut Gustave Roussy, 114 rue Edouard Vaillant, 94805 Villejuif, France; 5 Department of Medical Oncology, Centre Léon Bérard, 28 Promenade Léa et Napoléon Bullukian, 69008 Lyon, France; 6 Department of Medical Oncology, Jean Minjoz University Hospital, 3 Boulevard Alexandre Fleming, 25030 Besançon, France; 7 Department of Medical Oncology, Institut de Cancérologie de l’Ouest, Site Hospitalier Nord Boulevard Jacques Monod, 44805 Saint-Herblain, France; 8 Department of Medical Oncology, Institut Curie, 26 rue d’Ulm, 75248 Paris, France; 9 Department of Medical Oncology, Institut Bergonié, 229 cours de l’Argonne, 33000 Bordeaux, France; 10 Department of Medical Oncology, Centre Alexis Vautrin, 6 Avenue de Bourgogne, 54519 Vandœuvre-lès-Nancy, France; 11 Department of Medical Oncology, Centre Oscar Lambret, 3 Rue Frédéric Combemale, 59000 Lille, France

**Keywords:** Sarcoma, Malignant solitary fibrous tumor, Trabectedin, Growth modulation index

## Abstract

**Background:**

Advanced malignant solitary fibrous tumors (SFTs) are rare soft-tissue sarcomas with a poor prognosis. Several treatment options have been reported, but with uncertain rates of efficacy. Our aim is to describe the activity of trabectedin in a retrospective, multi-center French series of patients with SFTs.

**Methods:**

Patients were mainly identified through the French RetrospectYon database and were treated between January 2008 and May 2013. Trabectedin was administered at an initial dose of 1.5 mg/m^2^, q3 weeks. The best tumor response was assessed according to the Response Evaluation Criteria In Solid Tumors 1.1. The Kaplan–Meier method was used to estimate median progression-free survival (PFS) and overall survival (OS). The growth-modulation index (GMI) was defined as the ratio between the time to progression with trabectedin (TTP_n_) and the TTP with the immediately prior line of treatment (TTP_n-1_).

**Results:**

Eleven patients treated with trabectedin for advanced SFT were identified. Trabectedin had been used as second-line treatment in 8 patients (72.7 %) and as at least third-line therapy in a further 3 (27.3 %). The best RECIST response was a partial response (PR) in one patient (9.1 %) and stable disease (SD) in eight patients (72.7 %). Disease-control rate (DCR = PR + SD) was 81.8 %. After a median follow-up of 29.2 months, the median PFS was 11.6 months (95 % CI = 2.0; 15.2 months) and the median OS was 22.3 months (95 % CI = 9.1 months; not reached). The median GMI was 1.49 (range: 0.11–4.12).

**Conclusion:**

Trabectedin is a very promising treatment for advanced SFTs. Further investigations are needed.

## Background

Solitary fibrous tumors (SFTs) are rare soft-tissue sarcomas with an estimated incidence of < 0.1/100,000/year [1]. Initially described in pleura, it is now established that SFTs can occur in almost any extra-pleural site. They develop mainly in people aged 50–70 years, with a gender ratio of 1:1.

Solitary fibrous tumors constitute a heterogeneous group of rare spindle-cell tumors, with an unpredictable course. Morphologically, SFTs are characterized by a combination of alternating hypocellular and hypercellular areas, separated by hyalinized collagen, with hemangiopericytoma-like vessels. It should be noted that hemangiopericytomas (initially described as a distinct neoplasm of pericytic origin) and SFTs actually constitute a single entity with a morphological continuum [2].

A large majority of cases are benign neoplasms which have an excellent outcome after complete surgical resection [3]. However, a small proportion of cases follow an aggressive course (recurrence after local treatment or metastatic progression). Increased mitotic activity (≥4 mitoses per 10 high-power fields), greater cellularity, cellular polymorphism, and the presence of necrosis are associated with malignant behavior [4].

The standard treatment for localized SFTs is complete surgical resection. No consensus exists on the treatment of advanced stages of this disease. Anthracycline-based chemotherapy or dacarbazine achieve variable response rates, ranging from 0 to 50 % [5–8]. In addition, interesting results have been reported in small retrospective series of patients who had received temozolomide plus bevacizumab [9] or tyrosine kinase inhibitors (sunitinib [10], sorafenib [11], pazopanib [12], and imatinib [13]).

Trabectedin (Yondelis(®) [PharmaMar S.A., Madrid, Spain]) is one of the most promising agents to have been developed over the last two decades, and is now an approved treatment for patients with anthracycline/ifosfamide-resistant soft-tissue sarcoma and for those unsuited to these treatments [14, 15]. Objective responses have already been documented in two case reports [16, 17].

This retrospective study describes the efficacy and toxicity of trabectedin in advanced SFTs.

## Methods

### Patients

Most patients were identified through the French RetrospectYon database, which collected data from patients treated with trabectedin between January 2008 and December 2011 in French Sarcoma Group centers. Additional patients were identified through the electronic databases of certain French cancer centers between January 2012 and May 2013.

We collected data on: patients’ characteristics at diagnosis; disease characteristics including primary tumor site, extent of disease and pathological features; previous chemotherapy; and clinical outcomes with trabectedin therapy.

This study was approved by the ethics committee of the French Sarcoma Group as well as the scientific board (which permitted access to the RestrospectYon database). Access to local databases was granted by the institutional scientific board of each participating center belonging to the French Sarcoma Group. All patients consented in writing to the anonymized assessment and analysis of clinical data and therapeutic outcome.

### Pathology

Diagnosis was established by pathologists at each French Sarcoma Group center, in accordance with the World Health Organization classification and criteria [18]. Immunologic profile was systematically assessed using CD34 and other antibodies, depending on histopathological profile.

Tumors were scored according to mitotic index, cellularity, cytonuclear polymorphism, and necrosis. The mitotic index was determined using 10 high-power fields (×400); necrosis was assessed with a two-area scale (<50 % or >50 %). Tumor size was also measured.

### Treatment

Following the French consensus, trabectedin was given at the approved initial dose of 1.5 mg/m^2^ as a 24-h continuous i.v. infusion every three weeks, along with anti-emetic prophylaxis and a granulocyte colony-stimulating factor. Dose reductions (from 1.5 to 1.2 mg/m^2^, and then to 1 mg/m^2^) were decided in cases of toxicity. The standard number of cycles in patients with disease control was six, and maintenance therapy was an option (depending on clinical benefit and the absence of toxicity) at the discretion of the physician.

### Response assessment

#### Radiological assessment

Disease status was retrospectively assessed from a CT scan at baseline and every 2–3 months. Response to treatment was judged by applying the Response Evaluation Criteria in Solid Tumors (RECIST 1.1) [19]. Disease control rate (DCR) was defined as complete response (CR) + partial response (PR) + stable disease (SD). The clinical benefit rate was defined as DCR ≥ 6 months [10].

#### Growth-modulation index (GMI)

The growth-modulation index (GMI) is a concept introduced in 1998 by Von Hoff [20, 21] to compare successive times to progression (TTP) in an individual patient. It is defined as the ratio between the TTP with an experimental treatment (TTP_n_) and the TTP with the immediately prior line of treatment (TTP_n-1_). Because TTPs tend to be shorter with each treatment line, it has been proposed that a GMI >1 or even >1.33 could be a surrogate for drug activity in phase II trials.

### Statistical analyses

The data are presented using frequency and percentage for categorical variables and median and range for continuous variables.

All survival times were calculated from the date of first administration of trabectedin, and were estimated by the Kaplan–Meier method using the following first-event definitions: progression according to RECIST or death for progression-free survival (PFS) and death from any cause for overall survival (OS). Patients who were still progression-free were censored at the time of their last follow-up. All statistical analyses were conducted using STATA 13.0 software.

## Results

### Patients

We identified eleven patients with progressive malignant SFT who were treated with trabectedin between January 2008 and May 2013. Median age at diagnosis was 55 years (range: 37–62 years). Patient and tumor characteristics are summarized in Table 1.

**Table 1:** Patient and disease characteristics

Characteristic	
Age (years) (median, range)	55 (37–62)
Gender	
Male	2 (18.2 %)
Female	9 (81.8 %)
ECOG performance status at diagnosis	
0	7 (63.6 %)
1	4 (36.4 %)
Primary tumor site	
Pleura	5 (45.5 %)
Abdomen	2 (18.2 %)
Pelvis	2 (18.2 %)
Ovary	1 (9.1 %)
Upper limb	1 (9.1 %)
Pathological characteristics at diagnosis	
Mitotic index (/10 HPF^a^) (median, range)	9 (3–30)
Necrosis	
No	6 (54.5 %)
Yes	5 (45.5 %)
<50 %	3
>50 %	0
Unknown	2
Tumor size (mm) (median, range)	145 (45–180)
Tumor classification at diagnosis	
Benign	1 (9.1 %)
Malignant	10 (90.9 %)
Stage of disease at diagnosis	
Local	9 (81.8 %)
Metastatic	2 (18.2 %)

^a^/10 high-power fields

### Treatment

All patients had received prior chemotherapy. Trabectedin was used as a second-line treatment in eight patients (72.7 %): six had previously received anthracycline-based chemotherapy and two a targeted therapy; it was at least third-line treatment in three patients (27.3 %). The best RECIST response to a prior therapy was a PR in one patient (9.1 %) and SD in seven (63.6 %).

All patients were metastatic and had disease progression at the start of trabectedin therapy (local progression: 63.6 %; distant progression: 100 %).

Patients received a median of nine cycles of treatment (range: 2–12). Median treatment duration was 6.1 months (range: 1–11.5 months). All patients received a standard dose of 1.5 mg/m^2^ except for one who was started on 1.2 mg/m^2^. Two patients (18.2 %) required dose reduction because of toxicity.

Reasons for stopping trabectedin were disease stabilization after at least six cycles (*n* = 5, 45.5 %), progressive disease (*n* = 4, 36.3 %), and toxicity (*n* = 2, 18.2 %) (Table 2).

**Table 2:** Previous treatments and trabectedin administration

Pt no.	Prior systemic treat. before trab.	Best RECIST response to systemic treatments	Reason for discontinuing therapy	Disease status just before starting trab.	Numb of trab. cycles	Dose decr.	Reason for stopping trab.
1^a^	A →	SD →	Stab.	PD (L + M)	10	Y	Stab.
	Soraf n°1 →	SD →	Stab.				
	Soraf. n°2 →	SD →	Stab.				
	Soraf. n°3 →	SD →	Progr.				
2	Soraf	PD	Progr.	PD (M)	9	N	Stab.
3	AI	PD	Progr.	PD (L + M)	6	N	Progr.
4	A	PR	Stab.	PD (L + M)	12	Y	Tox.
5	AI	SD	Stab.	PD (M)	11	N	Stab.
6	Ima →	SD →	Prog.	PD (L + M)	12	N	Stab.
	AD →	SD →	Stab.				
	Cy →	SD →	Progr.				
	Soraf. →	PD →	Tox.				
7	A →	SD →	Stab.	PD (L + M)	2	N	Progr.
	Cy →	PD →	Progr.				
	Suni →	SD →	Progr.				
	Bev + Tmz →	SD →	Progr.				
8	MAID	SD	Stab.	PD (M)	4	N	Tox.
9	AI	SD	Progr.	PD (M)	3	N	Progr.
10	AD	SD	Stab.	PD (L + M)	6	N	Stab.
11	PLK-Inh.	PD	Progr.	PD (L + M)	11	N	Progr.

*L* local, *M* metastatic, *trab*. trabectedin, *dose decr*. dose decrease, *A* adriamycin, *I* ifosfamide, *D* dacarbazine, *M* Mesna, *Cy* cyclophosphamide, *Soraf* sorafenib, *Ima* imatinib, *Suni* sunitinib, *Bev + Tmz* bevacizumab + temozolomide, *PLK-Inh* Polo-Like Kinase inhibitor, *SD* stable disease, *PD* progressive disease, *Y* yes, *N* no, *Stab* disease stabilization, *Progr* disease progression, *Tox* toxicity

^a^Patient 1 received sorafenib three times, in three successive lines of treatment

### Clinical outcomes after trabectedin therapy

Data are presented in Table 3.

**Table 3:** Clinical outcomes after trabectedin therapy

Pt no.	Best RECIST response	PFS (months)	Progression	OS (months)	Status at last follow-up	GMI = TTP2/TTP1		
						TTP1 (months)	TTP2 (months)	GMI
1	SD	11.7	N	11.7	Alive	4.9	-	-
2	SD	11.6	Y	29.2	Alive	5.6	11.6	2.07
3	SD	3.8	Y	9.1	Dead	1.9	3.8	2.02
4	SD	14.6	Y	22.3	Dead	12.6	14.6	1.16
5	SD	30.9	N	30.8	Alive	7.1	-	-
6	SD	11.0	Y	20.9	Alive	3.1	11.0	3.61
7	PD	1.9	Y	7.0	Dead	18.7	1.9	0.11
8	PR	7.5	Y	23.4	Dead	5.1	7.5	1.49
9	PD	2.0	Y	11.1	Dead	7.7	2.0	0.26
10	SD	15.2	Y	17.7	Dead	25.3	15.2	0.6
11	SD	12.3	Y	74.2	Alive	3.0	12.3	4.12
*Median*		*11.6*		*22.3*		*5.6*	*11.6*	*1.49*

*SD* stable disease, *PR* partial response, *PD* progressive disease, *GMI* growth modulation index, *TTP1* time to progression with the prior last-line treatment, *TTP2* time to progression with trabectedin

#### Response

PR was observed in one patient (9.1 %) and SD in eight patients (72.7 %). The DCR was 81.8 %. Except for one patient, SD and PR were confirmed at 6 months. The clinical benefit rate was thus 72.7 %.

Among the eight patients who received trabectedin as a second-line treatment, PR was observed in one patient (12.5 %) and SD in six patients (75 %), with a DCR of 87.5 %.

The median follow-up was 29.2 months (95 % confidence interval [CI] = 11.7 months; not reached).

**Fig. 1:**
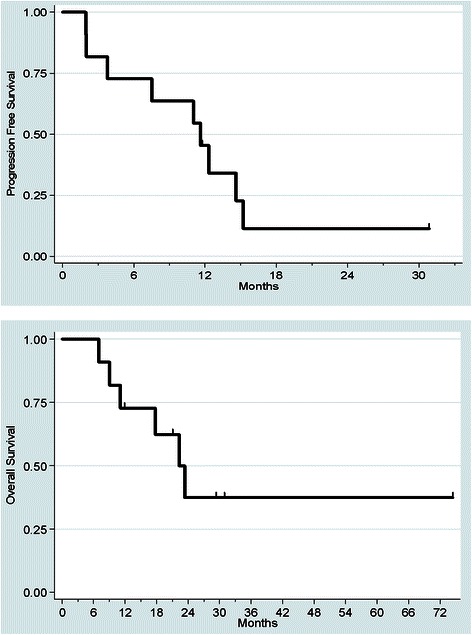
Kaplan-Meier curves for progression-free and overall survival from the start of trabectedin therapy

The median PFS was 11.6 months (95 % CI = 2.0; 15.2 months) and the median OS was 22.3 months (95 % CI = 9.1 months; not reached). The 3- and 6-month PFS were respectively 81.8 % (95 % CI = 44.8; 95.1 %) and 72.7 % (95 % CI = 37.1; 90.3 %). The 12- and 24-month OS rates were respectively 72.7 % (95 % CI = 37.1; 90.3 %) and 37.4 % (95 % CI = 9.5; 66.3 %) (Fig. 1). At the time of analysis, five patients were alive (45.6 %) and only one had progressive disease.

#### GMI

**Fig. 2:**
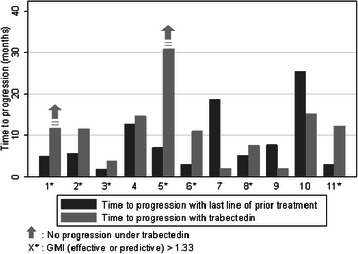
Comparison of time to progression with trabectedin (TTP2) versus time to progression with prior therapy (TTP1) for the 11 patients. The growth modulation index (GMI) is the ratio between TTP2 and TTP1. Two patients did not progress under trabectedin (patient 1 and patient 5), so that only a minimum predictive GMI could be estimated. All in all, seven patients had a GMI (effective or predictive) > 1.33 (63.6 %, 95 % CI = [30.8–89.1 %])

The median TTP with the last-line treatment before trabectedin (median TTP1) was 5.6 months (range: 1.9–25.3 months). The median growth modulation index was 1.49 (range: 0.11–4.12). For four patients, the GMI was higher than 2. Data were missing for two patients who had not progressed after trabectedin but whose predictive GMI was also higher than 2. In total, seven patients had a GMI (actual or predictive) of >1.33 (63.6 %, 95 % CI = [30.8–89.1 %]) (Fig. 2).

#### Toxicity

Grade 3–4 toxicity with trabectedin was observed in only three patients (27.3 %), and was essentially hematologic and hepatic (elevation in hepatic transaminases > 5× ULN). Two patients (18.2 %) required a dose reduction and two patients had to stop trabectedin because of toxicity, but no toxicity-related deaths were observed.

## Discussion

This study is the first to specifically report on the efficacy of trabectedin in a relatively large sample of patients with an advanced malignant solitary fibrous tumor.

We found very interesting results with an overall response rate of 9.1 % and a DCR of 81.8 %. The clinical benefit rate was 72.7 %. However, it should be noted that there is no standard definition of the duration of DCR required to qualify as “clinical benefit” in retrospective studies. The median PFS of 11.6 months (95 % CI = 2.0; 15.2 months) and the median OS of 22.3 months (95 % CI = 9.1 months; not reached) were favorably compared with almost all previous results obtained using conventional chemotherapy or a targeted therapy (Table 4).

**Table 4:** Median PFS (by RECIST) and median OS with other treatments for advanced SFT

Reference	n =	Regimen	Front-line/ Further-line	Median f-u (months)	Median PFS (months)	Median OS (months)
Stacchiotti [6]	31	Anthracycline-based chemo.	25/6	-	4 (range : 2–15) (front-line : 4)	11.5 (range : 3–50)
	19	High dose ifosfamide single agent	11/8	-	3 (range : 2–9) (front-line : 3 (range : 2–9))	11 (range : 3–50)
Park [5]	21^a^	Conventional chemo. (anthracycline-based/gemcitabine-based/paclitaxel : 15/5/5)	18/7	-	4.6 (95 % CI = 3.7–5.6) (front-line: 4.6 (95 % CI = 4–5.3))	22.8 (95 % CI = 3.1–42.6)
Stacchiotti [7]	8	Dacarbazine	0/8	-	7 (range : 2–12)	-
Park [9]	14	Bevacizumab + temozolomide	9/5	34	10.8 (95 % CI = 8.13-not reached)	24.3
Stacchiotti [10]	35	Sunitinib	10/25	-	6 (95 % CI = 4.03–8.01)	16 (95 % CI = 12.07–25.9)
Valentin [11]	5	Sorafenib	0/5	-	-	19.7

*f-u* follow-up, *PFS* progression-free survival, *OS* overall survival

^a^Of 21 patients, 4 received more than one regimen of chemotherapy, for a total of 25 treatments

The strength of this work was the use of the Growth-Modulation Index, a relevant parameter which takes into account the natural course of solitary fibrous tumors. Based on the fact that SFTs are usually slowly progressive diseases, and the fact that TTPs tend to be shorter with successive lines of treatment, a high GMI strongly suggests drug activity.

Cousin et al. showed that GMI was strongly correlated with OS in pre-treated advanced soft-tissue sarcomas [22]. More recently, Penel et al. have shown that a GMI >1.33, as reported in our study, was strongly correlated with improved PFS and even OS in advanced soft-tissue sarcoma patients receiving trabectedin as a salvage therapy [23].

Interestingly, we found long-lasting responses with trabectedin, even with non-indolent disease. Indeed, two patients had a long-lasting response in disease that had been rapidly progressive before introducing trabectedin (PFS of 11.0 months and 12.3 months after trabectedin, with a GMI of 3.61 and 4.12, respectively), thus highlighting the impact of trabectedin on the natural course of this disease. A further patient had non-progressive disease for 30 months after starting trabectedin therapy (which lasted 9 months) despite presenting with disease progression only 7 months after starting anthracycline-based chemotherapy. In this patient, the predictive GMI was at least 4.28.

Several sources of data suggest the antitumor activity of trabectedin in translocation-related sarcoma. This has been clearly established in the case of myxoid round-cell liposarcoma, where the characteristic chromosomal translocations of t(12;16)(q13;q11) and t(12;22)(q13;q12) code for the fusion transcripts *FUS-DDIT3* and *EWS-DDIT3*, which bind to specific DNA and act as transcription factors for oncoproteins [24]. It has been shown that the antitumor activity of trabectedin in myxoid round-cell liposarcoma was correlated with the expression of the fusion transcript FUS-DDIT3 by interfering with the DNA-binding site of the transcript and by then displacing the transcription factors [25]. Extrapolation of such mechanisms led to the hypothesis that other translocation-related sarcomas (TRS) that express other fusion transcripts may have a particular sensitivity to trabectedin. Thus Le Cesne et al. have provided results from a retrospective pooled analysis of 81 patients with TRS treated with trabectedin in eight phase II trials. They reported an overall control rate of 59 %, a median PFS of 4.1 months, and a median OS of 17.4 months [26]. Promising results have also been reported among TRS patients pretreated with conventional chemotherapy in a randomized phase II trial [27].

As regards SFT of the pleura specifically, a recent study assessed the multimodal management of such tumors (all stages included). Twenty-six of the 68 patients had recurrence after first-line treatment. Nine received trabectedin (as a second- or third-line treatment) with a disease control rate of 78 % and a median TTP of 3.4 months [28]. However, responses were not confirmed at 3 months and, above all, no data were available for TTP on the previous line of treatment. Therefore, in contrast to our study, it is not possible to take into account the natural course of the disease.

Lastly, recent data have highlighted *NAB2–STAT6* fusion, which is a result of inversion within chromosome 12, as a distinct molecular feature of SFTs [29, 30]. In the context of SFTs, *NAB2* gains an activation domain from the signaling molecule STAT6, which converts a transcriptional repressor (*NAB2*) into a potent transcriptional activator (*NAB2–STAT6*) of *EGR1* (Early Growth Response 1), a zinc-finger transcription factor. This leads to the constitutive activation of EGR-mediated transcription target genes such as *IGF2*, *FGF2*, *PDGFD*, or *FGFR*, which are implicated in the differentiation or proliferation pathways.

By analogy with myxoid liposarcoma, we can hypothesize that trabectedin interferes with the physical interaction of the NAB2–STAT6 fusion protein with EGR1 to ensure its specific anti-tumor activity in SFTs. However, no experimental data exist to suggest this specific effect of trabectedin in SFTs.

Even though an important gap remains between progress in diagnosis and advances in therapy, we are now better able to relate specific sarcoma subtypes to specific treatments [14, 31–34]. Trabectedin could become standard of care in the narrow field of advanced SFTs.

## Conclusion

Our data suggest that trabectedin is a very promising systemic treatment for malignant solitary fibrous tumors and should be strongly considered as an option along with other evidence-based therapies such as anthracyclines, dacarbazine, bevacizumab + temozolomide, sunitinib, and pazopanib.

However, we need further clinical investigations along with the experimental data associated with dedicated phase-II trials to understand the mechanisms of action of trabectedin in this rare sarcoma subtype and to validate this treatment prospectively in larger series of patients.

## Abbreviations

DCR: Disease-control rate
GMI: Growth-modulation index
OS: Overall survival
PD: Progressive disease
PR: Partial response
RECIST: Response evaluation criteria in solid tumors
SD: Stable disease
TTP: Time to progression
